# Role of Protein Phosphatase 2A in Osteoblast Differentiation and Function

**DOI:** 10.3390/jcm6030023

**Published:** 2017-02-23

**Authors:** Hirohiko Okamura, Kaya Yoshida, Hiroyuki Morimoto, Jumpei Teramachi, Kazuhiko Ochiai, Tatsuji Haneji, Akihito Yamamoto

**Affiliations:** 1 Department of Histology and Oral Histology, Institute of Biomedical Sciences, Tokushima University Graduate School, 3-18-15, Kuramoto, Tokushima 770-8504, Japan; jumptera@tokushima-u.ac.jp (J.T.); tat-hane@tokushima-u.ac.jp (T.H.); akihito@tokushima-u.ac.jp (A.Y.); 2 Department of Oral healthcare educations, Institute of Biomedical Sciences, Tokushima University Graduate School, 3-18-15, Kuramoto, Tokushima 770-8504, Japan; Kaya@tokushima-u.ac.jp; 3 Department of Anatomy, School of Medicine, University of Occupational and Environmental Health, 1-1, Iseigaoka, Yahatanishi, Kitakyushu, Fukuoka 807-8555, Japan; morimoto@med.uoeh-u.ac.jp; 4 Department of Veterinary Nursing and Technology, School of Veterinary Science, Nippon Veterinary Nursing and Life Science University, Tokyo 180-8602, Japan; kochiai@nvlu.ac.jp

**Keywords:** protein phosphatase, bone formation, osteoblast differentiation, osteosarcoma cells, osteoclastogenesis, adipogenesis

## Abstract

The reversible phosphorylation of proteins plays hugely important roles in a variety of cellular processes, such as differentiation, proliferation, and apoptosis. These processes are strictly controlled by protein kinases (phosphorylation) and phosphatases (de-phosphorylation). Here we provide a brief history of the study of protein phosphorylation, including a summary of different types of protein kinases and phosphatases. One of the most physiologically important serine/threonine phosphatases is PP2A. This review provides a description of the phenotypes of various PP2A transgenic mice and further focuses on the known functions of PP2A in bone formation, including its role in osteoblast differentiation and function. A reduction in PP2A promotes bone formation and osteoblast differentiation through the regulation of bone-related transcription factors such as Osterix. Interestingly, downregulation of PP2A also stimulates adipocyte differentiation from undifferentiated mesenchymal cells under the appropriate adipogenic differentiation conditions. In osteoblasts, PP2A is also involved in the ability to control osteoclastogenesis as well as in the proliferation and metastasis of osteosarcoma cells. Thus, PP2A is considered to be a comprehensive factor in controlling the differentiation and function of cells derived from mesenchymal cells such as osteoblasts and adipocytes.

## 1. History of Protein Phosphorylation and De-Phosphorylation

Protein phosphorylation plays a cardinal role in regulating many cellular processes in eukaryotes. Protein phosphorylation is the major currency of signal transduction pathways; the specific level of phosphorylation of any given signal transduction protein is reversibly controlled by protein kinases and protein phosphatases. The study of protein phosphorylation and de-phosphorylation has a long history. At the beginning of the 1900s, studies concerning the physicochemical properties of canonical phosphoproteins such as casein in milk and vitellin were first performed.

Later on, seminal work on the activity of muscle phosphorylase led to the concept of protein phosphorylation as a means of regulating enzyme activity. Muscle phosphorylase exists in two forms, phosphorylase *a* and phosphorylase *b*. The enzyme is regulated by AMP; phosphorylase *a* retains 60% to 70% of its maximal activity in the absence of AMP, whereas phosphorylase *b* requires AMP for its activity. However, during the enzymatic conversion of phosphorylase *a* to *b*, no release of AMP was observed; furthermore, AMP could not be detected in phosphorylase *a* [1]. Later, the conceptual idea of phosphorylation as a means of controlling enzyme activity was discovered through the recognition of a dual requirement for ATP and a converting enzyme (named phosphorylase kinase) for the in vitro conversion of phosphorylase types [2,3,4,5,6]. This conversion included the transfer of a phosphate group from ATP to phosphorylase. Phosphorylase *a* is a phosphoprotein and the conversion of active phosphorylase to its inactive form was shown to involve enzymatic de-phosphorylation of the protein.

## 2. Kinases and Phosphatases

Since these initial discoveries, many studies have shown that reversible phosphorylation of proteins, orchestrated by the interplay of kinases and phosphatases, regulates a majority of the important signaling pathways in all living organisms. In fact, the reversible phosphorylation of proteins represents a fundamental mechanism used by all eukaryotic organisms, with up to 30% of all proteins being phosphorylated at any given time [7]. Protein phosphorylation and de-phosphorylation occur at three hydroxyl-containing amino acids, namely serine (Ser), threonine (Thr), and tyrosine (Tyr) residues. A proteomic study has revealed that 2244 human proteins contain phosphoserine (86.4%), phosphothreonine (11.8%), and phosphotyrosine (1.8%) at their 6600 phosphorylation sites [8]. Complete sequencing of the human genome revealed 518 putative protein kinases [9,10,11], which can be divided into 90 Tyr kinases (PTKs) and 428 Ser/Thr kinases (PSKs). The balanced specificity and reversibility of protein phosphorylation and de-phosphorylation appears to be conducted by similar numbers of protein kinases and phosphatases. However, intriguingly, while there are 107 putative protein Tyr phosphatases (PTPs) [12], there is a much lower number of Ser/Thr phosphatases (PSPs) (~30). While the numbers of PTKs and PTPs are roughly equal, the number of PSP subunits is an order of magnitude lower than the number of PSKs. This difference can be explained by the fact that there is a combinatorial formation of PSP holoenzymes from a shared catalytic subunit coupled with a large number of regulatory subunits.

PSPs are further classified into three major groups: (1) the aspartate-based phosphatases; (2) metal-ion-dependent phosphatases; and (3) phosphoprotein phosphatases [7,13]. PSPs dephosphorylate a broad range of phosphorylated Ser/Thr residues in proteins. PSPs consist of multi-subunit complexes including a scaffold subunit, a catalytic subunit, and a number of regulatory subunits. Each catalytic subunit displays catalytic specificity by binding to various different types of regulatory subunits. PSPs are further classified into six sub-groups, termed protein phosphatase 1 (PP1), PP2A, PP2B (also known as calcineurin or PP3), PP4, PP5, and PP6. In this review, we focus on the role of PP2A and its role in bone formation and osteoblast differentiation and function.

## 3. Structure of PP2A

PP2A is one of the most important serine/threonine phosphatases, and is essential for embryonic development, cell proliferation, and apoptosis. PP2A has been reported to occupy ~1% of the total amount of protein in mammalian cells [14]. PP2A is a heterotrimeric complex, and its enzymatic specificity and cellular localization are thought to be dependent on the nature of the regulatory subunit [15]. The central core dimer of PP2A is a 65 kDa scaffolding A subunit and a 36 kDa catalytic C subunit. Although the A and C subunits each have two highly homologous isoforms (Aα/β or Cα/β), the Aα and Cα isoforms are much more abundant than the β isoforms. On the other hand, there are many types of different PP2A regulatory subunits and they are subdivided into four distinct families: B (PR55), B′ (B56 or PR61), B″ (PR72), and B′′′ (PR93/PR110). Each of these subfamilies has been reported to have at least 16 members [16,17]. Variability within the B subunit is further increased by the existence of splice variants. The diverse composition of the PP2A holoenzyme provides many possibilities for cellular regulation. One of the most abundant cytosolic proteins, the PP2A catalytic (or C) subunit, can account for 0.1% of the total cellular protein in certain cell types [18]. To form an active phosphatase, the PP2A C subunit forms a dimeric core by binding to the scaffold A subunit, which is then selectively associated with one of a large number of regulatory B subunits. Therefore, eukaryotic cells can express a broad range of these PP2A complexes depending on the conditions. PP2A has been shown to have important roles in development, cell growth, and proliferation; in addition, suppression of the catalytic or regulatory subunit has been reported to induce apoptosis in some cells [19,20,21,22,23]. A small fraction of PP2A C can also exist as a heterodimer by binding to other proteins including the α4 subunit. Recently, a high-density mass spectrometry analysis revealed that additional proteins beyond B subunits can interact with the core dimer to form other heterotrimers [24]. The diversity of PP2A therefore derives from the fact that cells can assemble over 200 biochemically distinct complexes containing different combinations of A, B, and C subunits. The discovery of the potent inhibitor okadaic acid (OA) provides an invaluable tool for research on the role of serine/threonine protein phosphatases in intracellular signaling and cell function [25]. OA was originally discovered and isolated from the marine sponge *Halichondria okadaii* [26,27]. OA inhibits both PP2A and PP1 by interacting with the active core site of the catalytic subunit [13,28]. A structural analysis has revealed that the hydrophobic cage in the PP2A catalytic subunit is larger than in PP1, providing a potential explanation as to why OA is 100-fold more potent against PP2A than against PP1 [13].

## 4. Phenotype of PP2A C Subunit-Transgenic Mice

Numerous transgenic and knockout approaches have been used to address the function of the PP2A C subunit. Complete loss of PP2A Cα leads to early embryonic lethality at E6.5 in mice [19], although embryos develop normally until post-implantation at approximately E5.5–6.0. PP2A Cα–null embryos can form the primary ectoderm and endoderm but not the mesoderm [29], suggesting that PP2A Cα is indispensable for cell development, differentiation, and proliferation of the mesoderm layer.

PP2A impairment has been demonstrated to be relevant to Alzheimer’s disease (AD). In transgenic mice expressing a dominant-negative PP2A C subunit mutant (L199P), the tau protein is hyper-phosphorylated at Ser202/Thr205 and Ser422 [30]. This phosphorylation of tau at Ser202/Thr205 also occurred in mice expressing another dominant negative PP2A C subunit mutant (L309A) [31]. Hyper-phosphorylation and somato-dendritic accumulation of tau, as well as a decrease in PP2A expression, are observed in the brain of patients with AD, suggesting that a reduction of PP2A activity could possibly be involved in the pathogenesis in AD [32].

Hepatic fibrosis is a pathogenic response of the liver that occurs as a result of chronic injury and results in the deposition of large amounts of extracellular matrix (ECM) proteins [33]. Activation of α-smooth muscle actin-positive fibroblasts, derived from hepatic stellate cells (HSCs), is thought to be primarily responsible for ECM production during liver fibrosis [34]. TGF-β1/Smad signaling is thought to influence the fibrotic process by controlling hepatocyte proliferation and apoptosis as well as mediating the activation and ECM production of HSCs in response to liver injury [35,36]. Hepatocyte-specific ablation of PP2A Cα protects against CCl_4_-induced chronic liver injury and fibrosis and this protective effect is mediated, at least partially, through impaired TGF-β1/Smad signaling.

Systemic lupus erythematosus (SLE) is an autoimmune disease that arises due to T cell signaling defects. Increases in PP2A expression and activity in T cells, as well as increased IL-17 secretion, have been reported in patients with SLE [37,38,39]. Transgenic mice that overexpress PP2A Cα in T cells showed an increased susceptibility to immune-mediated glomerulonephritis in the absence of other immune defects [40]. Thus, PP2A Cα is thought to be involved in the pathogenesis of systemic lupus erythematosus (SLE) by promoting IL-17–mediated inflammation and facilitating the development of end-organ damage [40].

Cardiac hypertrophy is a pathological change that occurs in heart disease and it has been demonstrated to be associated with protein phosphatases. In particular, protein phosphatases, including PP2A, have been shown to be involved in cardiac structural remodeling by regulating the phosphorylation status of many cardiac proteins [41,42]. A cardiomyocyte-specific deletion of PP2A Cα was shown to cause cardiac hypertrophy and fibrosis along with a severe disruption of the Akt/GSK3β/β-catenin pathway, a signaling pathway that is important for regulating cardiomyocyte growth [43,44].

An important role of PP2A Cα was also reported in the epidermis of mammalian skin, which is a critical organ for maintaining body temperature and which protects animals against dehydration, mechanical stress, and infections [45]. Conditional knockout of PP2A Cα in the epidermis showed a disruption in both morphogenesis and the hair regeneration cycle in hair follicles [46]. PP2A is speculated to stimulate the Wnt signaling pathway in hair follicles and therefore a defect of PP2A Cα causes a failure in normal hair follicle formation. Furthermore, these mice exhibited decreased size and melanin deposition, as well as hyper-proliferation at the base of the claws.

PP2A Cα has also been shown to be an important factor for spermatogenesis and oocyte meiosis. Mice with a spermatocyte-specific deletion of PP2A Cα were infertile and showed a significant reduction in the size and weight of the testes [47]. Disordered spermatogonia and spermatocytes with a large number of vacuoles were also observed in these mutant testes [47]. Inactivation of PP2A Cα in mouse oocytes also caused female infertility [48]. Oocytes lacking PP2A Cα failed to complete the first meiotic division due to chromosome misalignment and abnormal spindle assembly, suggesting that PP2A Cα is essential for chromosome alignment and regulates the formation of the correct kinetochore-microtubule attachment [48].

Hepatic insulin resistance has been shown to be a critical feature of metabolic disorders such as obesity, type 2 diabetes, and coronary artery diseases [49]. Several reports have revealed that PP2A is involved in the metabolic actions of insulin. PP2A has been reported to be upregulated in insulin-resistant patients [50,51]. Liver-specific deletion of PP2A Cα resulted in improved glucose homeostasis without body weight and liver weight changes. These knockout mice showed enhanced glycogen deposition with increased serum triglycerides, cholesterol, low-density lipoprotein, and high-density lipoprotein, as well as increased insulin signaling and decreased expression of gluconeogenesis genes [52]. Based on the extent of all these findings it is very likely that further studies will uncover additional roles for PP2A Cα in various tissues under both physiological and pathological conditions.

## 5. Role of PP2A in Bone Formation and Osteoblast Differentiation

The effect of PP2A inhibition on bone formation and osteoblast differentiation was studied using OA, a potent inhibitor of PP2A. This compound stimulated alkaline phosphatase (ALP) activity in MC3T3-E1 cells, a mouse osteoblastic cell line [53]. OA also inhibited bone resorption stimulated by PTH, 1,25-dihydroxyvitamin D_3_, phorbol ester, or prostaglandin E_2_ [54]. Administration of OA to the calvarial region of mice resulted in increased bone mineral density, bone thickness, and mineral apposition in the injected region [55]. The ALP activity and the expression of bone-related genes, including Osterix, Bone sialoprotein (Bsp), and Osteocalcin (OCN), were also increased in MC3T3-E1 cells treated with OA, which led to an acceleration in osteoblast differentiation [55].

Many factors, including several transcription factors, regulate osteoblast commitment, differentiation, and function and, sequentially, bone formation. Osterix and Runx2 are essential transcription factors for osteoblast differentiation and bone formation [56]. Osterix belongs to the Sp family of transcription factors and it regulates the expression of a number of bone-related genes including Bsp and OCN [57,58,59,60]. The expression and activity of PP2A Cα were shown to be decreased in osteoblasts cultured with osteoblast differentiation medium [55]. PP2A Cα ablation itself induces osteoblast differentiation accompanied by an increase in ALP activity, and an increase in the expression of bone-related genes including Osterix, Bsp, and OCN, implying that PP2A is strongly correlated with osteoblast differentiation and mineralization [55]. In contrast, the abilities to differentiate and mineralize were suppressed in PP2A Cα–overexpressing cells [61]. This suppression of osteoblast differentiation was accompanied by a significant decrease in bone-related genes including Osterix, Runx2, Bsp, and OCN. Furthermore, exogenous Osterix expression resulted in a significant increase in Bsp and OCN expression in PP2A Cα–overexpressing cells. Thus, PP2A Cα is thought to be a negative regulator of Osterix expression since inhibition of PP2A leads to the induction of Osterix and osteoblast differentiation. PP2A is also involved in Bone morphogenetic protein (BMP)-Smad signaling, which is an important pathway for skeletal development, bone formation, and adult bone homeostasis [62]. PP2A binds to BMP receptors and de-phosphorylates Smads, leading to their nuclear translocation and the amplification of BMP-Smad signaling [63]. PP2A has also been reported to mediate oxidative stress–induced apoptosis in osteoblasts. Oxidative stress induces PP2A phosphatase activity and apoptotic cell death in osteoblasts, which is partly suppressed by the inhibition of PP2A activity [64]. In osteoblasts, Runx2 is phosphorylated by CDK1/cyclin B complexes during mitosis and this is reversed by PP2A after mitosis is completed; this is considered to be an important mechanism in maintaining the osteoblast phenotype [65]. The initial step in osteoblast adhesion is thought to be important for the development and improvement of biomaterials in both skeletal medicine and dentistry [66]. Osteoblast adhesion requires cytoskeleton rearrangement, which is mediated by a diverse set of proteins including Cofilin and RhoA [67]. Cell adhesion induces integrin activation, and Cofilin promotes the regeneration of actin filaments by severing pre-existing filaments. Since Cofilin is a substrate of PP2A, PP2A appears to regulate osteoblast adhesion through actin rearrangement [67]. PP2A thus shows broad functions in osteoblasts.

## 6. PP2A Cα in Adipocyte Differentiation

Adipocytes and osteoblasts are derived from common progenitor mesenchymal stem cells [68,69]. As such, the tightly controlled lineage commitment of mesenchymal stem cells has a critical role in the maintenance of bone homeostasis. Although many types of cells can be produced by mesenchymal stem cell differentiation, the commitment of mesenchymal stem cells to osteoblast and adipocyte lineages has been especially correlated with pathological and age-related abnormal bone remodeling [68,70,71]. The mechanisms involved in the commitment of mesenchymal stem cells to adipocytes have been widely studied and various key regulatory factors have been identified as being important. The commitment and differentiation of adipocytes is regulated by several transcription factors that also control the specific gene expression needed to acquire adipocyte function [72,73]. Chief among these genes is peroxisome proliferator-activated receptor γ (PPARγ), a master regulator of adipogenesis that directly stimulates CCAAT/enhancer binding protein α (C/EBPα) expression in the early stages of adipocyte differentiation [74,75]. Another important molecule is fatty acid-binding protein 4 (FABP4), which encodes a cytoplasmic fatty-acid binding protein that is involved in fatty acid trafficking in adipocytes [76]. In addition, adiponectin, an adipocyte-secreted cytokine, is important for whole body metabolism [77]. The expressions of FABP4 and adiponectin are themselves mediated by PPARγ and C/EBPα [78,79]. PP2A Cα is a critical factor in adipocyte differentiation because silencing of PP2A Cα was shown to stimulate adipocyte differentiation and lipid accumulation through the upregulation of these important adipocyte marker genes [80]. Moreover, PP2A Cα regulates adipocyte differentiation by regulating the Wnt/β-catenin signaling pathway [80]. Activation of Wnt signaling and accumulation of β-catenin stimulates osteoblastogenesis and suppresses adipogenesis by suppressing PPARγ and C/EBPα. In the absence of Wnt ligands, the cytoplasmic β-catenin protein is continuously phosphorylated by glycogen synthase kinase 3β (GSK-3β) and degraded by the ubiquitin-proteasome system [81,82]. Conversely, the binding of Wnt ligands to the cell surface receptors leads to the phosphorylation of GSK-3β and its inactivation, resulting in a failure to phosphorylate β-catenin [81,82]. Thus, the stabilized β-catenin translocates to the nucleus and mediates the expression of Wnt-targeted genes. Knockdown of PP2A Cα decreased Wnt10b expression and increased the activated form of GSK-3β, resulting in a reduction in the expression and transcriptional activity of β-catenin [80]. Knockdown of PPARγ and inhibition of GSK-3β reduced the accelerated adipogenesis in PP2A Cα–knockdown cells [80]. Thus, PP2A Cα is considered to be a critical factor for adipocyte differentiation through the Wnt/GSK-3β/β-catenin pathway and PPARγ expression.

## 7. Role of PP2A Cα in Osteoblast on Osteoclastogenesis

In the skeletal system, bone is continuously rearranged through the removal of old bone and the apposition of new bone tissue, processes which are mediated by osteoclasts and osteoblasts [83,84]. RANKL is a transmembrane molecule found on the surface of osteoblasts, which is occasionally also secreted as soluble RANKL (sRANKL). The receptor for RANKL (RANK) is expressed on the surface of osteoclast precursor cells and binding of RANKL (or sRANKL) to RANK leads to osteoclastogenesis. In contrast, binding of the decoy receptor osteoprotegerin (OPG) to RANKL prevents RANKL-induced signaling to osteoclast precursor cells and inhibits osteoclast differentiation. Thus, changes in the ratio of RANKL/sRANKL to OPG regulate bone metabolism by controlling the balance of osteoclast differentiation. Silencing of PP2A Cα in osteoblasts was shown to decrease RANKL and increase OPG expression and consequently there was a large decrease in the sRANKL/OPG ratio in the culture medium of these cells [85]. PP2A Cα expression in osteoblasts has been reported to play a pivotal role in osteoclastogenesis via the regulation of NFATc1-related gene expression [85].

## 8. PP2A Cα in Osteosarcoma

Osteosarcoma is the predominant form of malignant bone cancer, occurring mostly in children [86,87]. Despite progress in chemotherapy for osteosarcoma, tumor metastasis is a major cause of mortality. Osteosarcoma arises from mesenchymal bone-forming cells and mainly occurs in the long bones, such as the distal femur, the proximal tibia, and the humerus [88]. The molecular mechanisms underlying osteosarcoma formation are related to a complex karyotype and multiple genomic alterations [89,90]. Mutations of each PP2A subunit and decreases in PP2A activity in various cancer cells have been demonstrated numerous times and have been discussed in many other review articles [91,92,93]. Based on these findings, PP2A is considered to be a tumor suppressor protein. However, several observations suggest that suppression of PP2A activity is associated with other oncogenic changes and can induce transformation [94]. In fact, recent studies have demonstrated the complexity of PP2A function in several types of tumor cells such as pancreatic cancer cells, in which inhibition of PP2A activity actually suppresses growth and invasion of tumor cells [95,96,97]. These controversial roles for PP2A in tumor development might result from the fact that PP2A has many distinct subunits that allow PP2A to control different signaling pathways [98,99]. In the case of osteosarcoma, PP2A has been discovered to be involved in the ability to proliferate and metastasize. Increases in both PP2A activity and PP2A Cα expression were observed in malignant osteosarcoma tissues and osteosarcoma LM8 cells [100]. Downregulation of PP2A Cα in LM8 cells induced morphological changes and decreased the activation of NF-ĸB and FAK, which was followed by suppression of proliferation and migration in vitro [100]. In addition, a reduction in PP2A Cα in LM8 cells attenuates their ability to proliferate and metastasize in vivo [100]. Silencing of PP2A Cα was also shown to reduce the expression of the anti-apoptotic mitochondrial protein Bcl-2 and increase the sensitivity of osteosarcoma cells to serum deprivation-induced apoptosis [100]. PP2A thus appears to be an important factor regulating the proliferation and metastasis of osteosarcoma cells.

## 9. Conclusions and Future Directions

**Figure 1:**
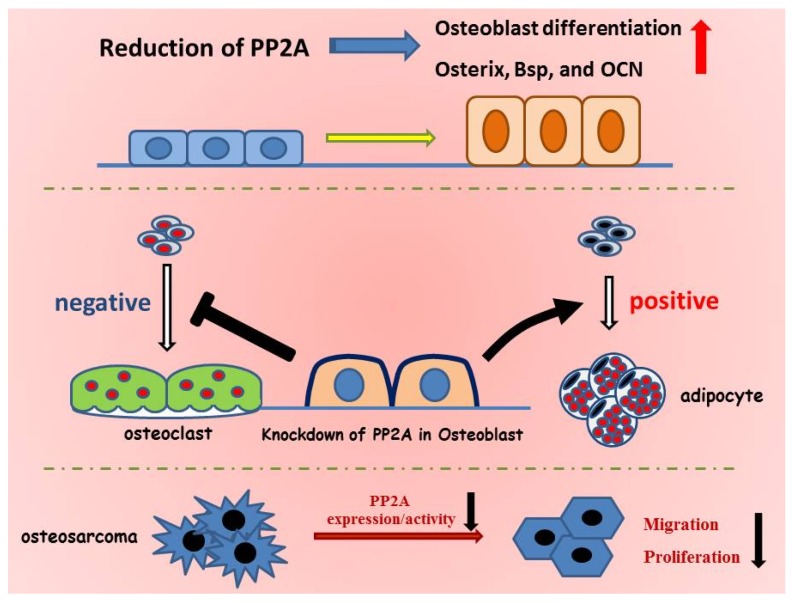
PP2A regulates osteoblast differentiation and function. PP2A is thought to have critical roles in not only osteoblast differentiation but also in the regulation of the differentiation of the surrounding cells such as adipocytes and osteoclasts. A reduction in PP2A accelerates osteoblast differentiation and bone formation through upregulation of the expression of bone-related genes including ALK phosphatase, Osterix, Bsp, and OCN. Downregulation of PP2A in osteoblasts negatively controls osteoclast differentiation by reducing the ratio of sRankL/OPG; in contrast, it positively regulates adipocyte differentiation by increasing the expression of adipocyte-related genes such as PPARγ and C/EBPα. Furthermore, greater expression of PP2A is observed in osteosarcoma cells and a reduction in PP2A suppresses the proliferation, migration, and metastasis of osteosarcoma cells.

In this review, we have provided a brief overview of protein kinases and phosphatases and then demonstrated that the phosphatase PP2A has a pivotal role in controlling bone formation, in differentiation of mesenchymal cells, and in the malignant properties of osteosarcoma cells, as shown in Figure 1. PP2A controls osteoblast and adipocyte differentiation through regulating the expression of transcription factors essential for establishing either the osteoblast or adipocyte phenotype. These findings suggest that PP2A has the ability to maintain the potential of mesenchymal stem cells. However, further studies are needed to understand the precise mechanism by which PP2A maintains this status. In addition, PP2A is involved in osteoblast function, including differentiation from osteoblast progenitor cells and the proliferation and metastasis of osteosarcoma cells. Further studies on PP2A will hopefully uncover more information about its broad function in osteoblasts and mesenchymal stem cells. PP2A is known to consist of several types of subunits, which exhibit unique subcellular localizations and functions. Moreover, PP2A can bind to a variety of partners through regulatory B subunit switching. During osteoblast differentiation, PP2A shows a unique pattern of expression and activity, implying that PP2A has different roles and partners at each differentiation stage. Therefore, further studies will be required to determine how PP2A subunits and their associations are involved in osteoblast differentiation and in the expression of bone-related genes. This information will hopefully lead to advances in a broad spectrum of applications including the development of bone regenerative therapies and osteosarcoma-targeting therapeutics.
